# Identification of ENO1 as a prognostic biomarker and molecular target among ENOs in bladder cancer

**DOI:** 10.1186/s12967-022-03509-1

**Published:** 2022-07-14

**Authors:** Zhengnan Huang, Yilin Yan, Tengjiao Wang, Zeyi Wang, Jinming Cai, Xiangqian Cao, Chenkai Yang, Fang Zhang, Gang Wu, Bing Shen

**Affiliations:** 1 Department of Urology, Tongji Hospital, School of Medicine, Tongji University, Shanghai, 200065 China; 2 Department of Urology, Shanghai General Hospital, Shanghai Jiaotong University School of Medicine, Shanghai, 200080 China; 3 Shanghai Key Lab of Cell Engineering, Shanghai, 200433 China; 4 Department of Stem Cells and Regenerative Medicine, Translational Medicine Research Center, Naval Medical University, Shanghai, 200433 China; 5 Department of Urology, Shanghai General Hospital Affiliated to Nanjing Medical University, Shanghai, 200080 China

**Keywords:** Bladder cancer, Enolase, Prognostic biomarker, Molecular target, Immune infiltration

## Abstract

**Background:**

Enolase is an essential enzyme in the process of glycolysis and has been implicated in cancer progression. Though dysregulation of ENOs has been reported in multiple cancers, their prognostic value and specific role in bladder cancer (BLCA) remain unclear.

**Methods:**

Multiple databases were employed to examine the expression of ENOs in BLCA. The expression of ENO1 was also validated in BLCA cell lines and tissue samples by western blotting and immunohistochemistry. Kaplan–Meier analysis, ROC curve, univariate and multivariate Cox regression were performed to evaluate the predictive capability of the ENO1. Gene ontology (GO) and Gene Set Enrichment Analyses (GSEA) analysis were employed to perform the biological processes enrichment. Function experiments were performed to explore the biological role of ENO1 in BLCA. The correlation of ENO1 with immune cell infiltration was explored by CIBERSORT.

**Results:**

By analyzing three ENO isoforms in multiple databases, we identified that ENO1 was the only significantly upregulated gene in BLCA. High expression level of ENO1 was further confirmed in BLCA tissue samples. Aberrant ENO1 overexpression was associated with clinicopathological characteristics and unfavorable prognosis. Functional studies demonstrated that ENO1 depletion inhibited cancer cell aggressiveness. Furthermore, the expression level of ENO1 was correlated with the infiltration levels of immune cells and immune-related functions.

**Conclusions:**

Taken together, our results indicated that ENO1 might serve as a promising prognostic biomarker for prognosticating prognosis associated with the tumor immune microenvironment, suggesting that ENO1 could be a potential immune-related target against BLCA.

**Supplementary Information:**

The online version contains supplementary material available at 10.1186/s12967-022-03509-1.

## Background

Bladder cancer (BLCA) is a heterogeneous malignant tumor [1]. There are about 500,000 new cases every year worldwide, posing a serious threat to human health [2]. To date, a variety of therapeutic methods, including surgical resection, chemotherapy, radiotherapy and immunotherapy, have been widely used in the treatment of BLCA [3, 4]. However, the overall curative effect is still not ideal enough to achieve a radical cure. Hence, it is of great clinical significance to make clear the mechanisms of cancer occurrence and progression, and to screen the pivotal factors for the early diagnosis and targeted therapy for BLCA.

Known as the Warburg effect, tumor cells preferentially choose the glycolytic pathway to provide energy for cell growth, even under aerobic conditions, rather than the more productive oxidative phosphorylation pathway [5, 6]. As the core energy metabolism characteristics of solid malignant tumors, Warburg effect is characterized by active glycolysis pathway, decreased oxygen consumption, increased glucose uptake rate, but less ATP production and significantly increased lactic acid production in metabolites [6]. Increasing studies have demonstrated that glycolytic enzymes such as enolases, play a pivotal role in cancer development [7–9]. Enolase is an essential enzyme in the process of glycolysis, catalyzing 2-phosphoglycerate into phosphoenolpyruvate [10, 11]. The three isoforms of ENO in mammalian cells include: ENO1, which is widely present in a variety of tissues [12, 13]; ENO2, which is mainly expressed in neurons and neuroendocrine tissues [12, 14]; and ENO3, which is expressed in muscle tissues [15, 16].

ENOs are multifunctional molecules that not only participate in glycolysis pathway and regulate energy metabolism homeostasis, but also have a hand in the occurrence and development of various tumors [17]. Recent reports have demonstrated that ENO1 plays a critical role in various tumor progression [18–22]. For example, ENO1 was overexpressed in gastric cancer and functioned as a potential carcinogen to promote tumor progression [23]. In addition, ENO2 was reported to be upregulated in BRAF V600E-mutated colorectal cancer and promote cells proliferation and migration [24]. In another study, ENO3 was identified as an effective clinical biomarker for its selective role in the development of targeted therapies against lung adenocarcinoma [25]. These findings indicated that the ENO isoforms have significant value in different types of cancer. Nonetheless, the association between ENO isoforms and the prognosis of patients with BLCA is rarely reported.

Herein, we utilized bioinformatics analysis tools to explore the expression and multilevel clinical value of ENOs in BLCA, and identified ENO1 as a promising immune-related target, providing a novel strategy for the diagnosis and clinical treatment of BLCA.

## Methods

### Expression of ENOs in BLCA

Oncomine database (https://www.oncomine.org/resource/login.html) and TIMER database (https://cistrome.shinyapps.io/timer/) were utilized to quest the expression of ENOs between multiple cancer tissues and corresponding adjacent normal tissues. GEO database (http://www.ncbi.nlm.nih.gov/geo; GSE13507) was also used to analyze the expression difference of ENOs between BLCA tissues and normal samples. The association of the ENOs expression and clinicopathologic parameters was explored in UALCAN (http://ualcan.path.uab.edu/), TCGA-BLCA (https://www.tcga-data.nci.nih.gov/tcga) and GEO (GSE13507 and GSE32894) databases.

### Clinical samples

Fresh BLCA tissues and adjacent non-tumorous tissues were acquired from the patients at the time of surgery from the Shanghai General Hospital. Formalin-fixed, paraffin-embedded BLCA tissues and correlative clinicopathological information were also collected from Shanghai General Hospital.

### Cell culture and transfection

The human BLCA cell lines 5637 and UMUC-3 were cultured in RPMI-1640 medium (Invitrogen) at 37 °C with 5% CO_2_. All media are supplemented with 10% FBS and penicillin/streptomycin. For the knockdown assay, small interfering RNAs targeting ENO1 (siENO1-1 and siENO1-2) were applied, and scramble siRNAs (siNC) as the negative control. The siRNA sequences targeting ENO1 were listed in Additional file 1: Table S1.

### RNA isolation and quantitative Real-Time PCR

Total RNA was isolated from the cultured cells using the TRIzol reagent (TaKaRa), RNA was then converted into cDNA by applying the Prime-Script RT-PCR kit (TaKaRa). The mRNA expression levels of genes were examined using SYBR Green in an ABI 7500 StepOne Plus Real-Time PCR instrument (Applied Biosystem). The specific primer sequences were listed in Additional file 1: Table S1.

### Western blotting and immunohistochemistry

Western blotting was performed according to the standard methods as previously described [26]. Primary antibodies against ENO1 (1:2000, 11,204–1-AP, Proteintech) and GAPDH (1:1000, #5174, Cell Signaling Technology) were used. IHC staining of paraffin-embedded tissues with antibody against ENO1 (1:100, 11,204–1-AP, Proteintech) was performed following the standard procedures as previously described [27].

### Enrichment analysis of ENO1 co-expression network in BLCA

The stat packet of R software was employed to determine the co-expression genes associated with ENO1 expression in TCGA-BLCA. The clusterProfiler package of R software was utilized to perform GO function and KEGG pathway enrichment analysis of co-expressed genes.

### Gene set enrichment analysis

GSEA v4.1.0 software was applied to perform GSEA to investigate meaningful biological processes associated with ENO1 expression. Pathways with nominal p-value < 0.05 and FDR < 0.25 were considered significantly enriched.

### Prognostic analysis

Survival analysis was performed using the survival package and survminer package of R software to draw the Kaplan–Meier curves on BLCA samples. Survival package was also applied to perform univariate and multivariate Cox analysis, and survivalROC package was utilized to draw ROC curves.

### Cell proliferation assay

2,000 cells/well were seeded into a 96-well plate. If cells adhered to the bottom, 10 μL MTT was added to each well for 4 h at 37 °C and it was identified as 0 h. The formazan crystals were dissolved in dimethyl sulfoxide (DMSO) at 37 °C for 15 min and the absorbance at 490 nm was examined. After 24, 48, and 72 h, the similar procedure was performed.

### Transwell invasion assay

A total of 1 × 10^5^ cells were seeded into the top of an 8 μm pore-size Transwell chamber pre-coated with diluted Matrigel (BD Biosciences), then 500 μL medium containing 10% FBS was added to the bottom chamber. After the incubation for 24 h, cells were fixed in formaldehyde, stained with crystal violet, and counted by applying a microscope.

### Immune evaluation

CIBERSORT package of R software was used to detect the proportion of 22 immune cells in BLCA samples with low and high ENO1 expression, and Pearson’s correlation was assessed between the proportions and ENO1 expression.

## Results

### Transcriptional level of the ENOs in patients with BLCA

**Fig. 1:**
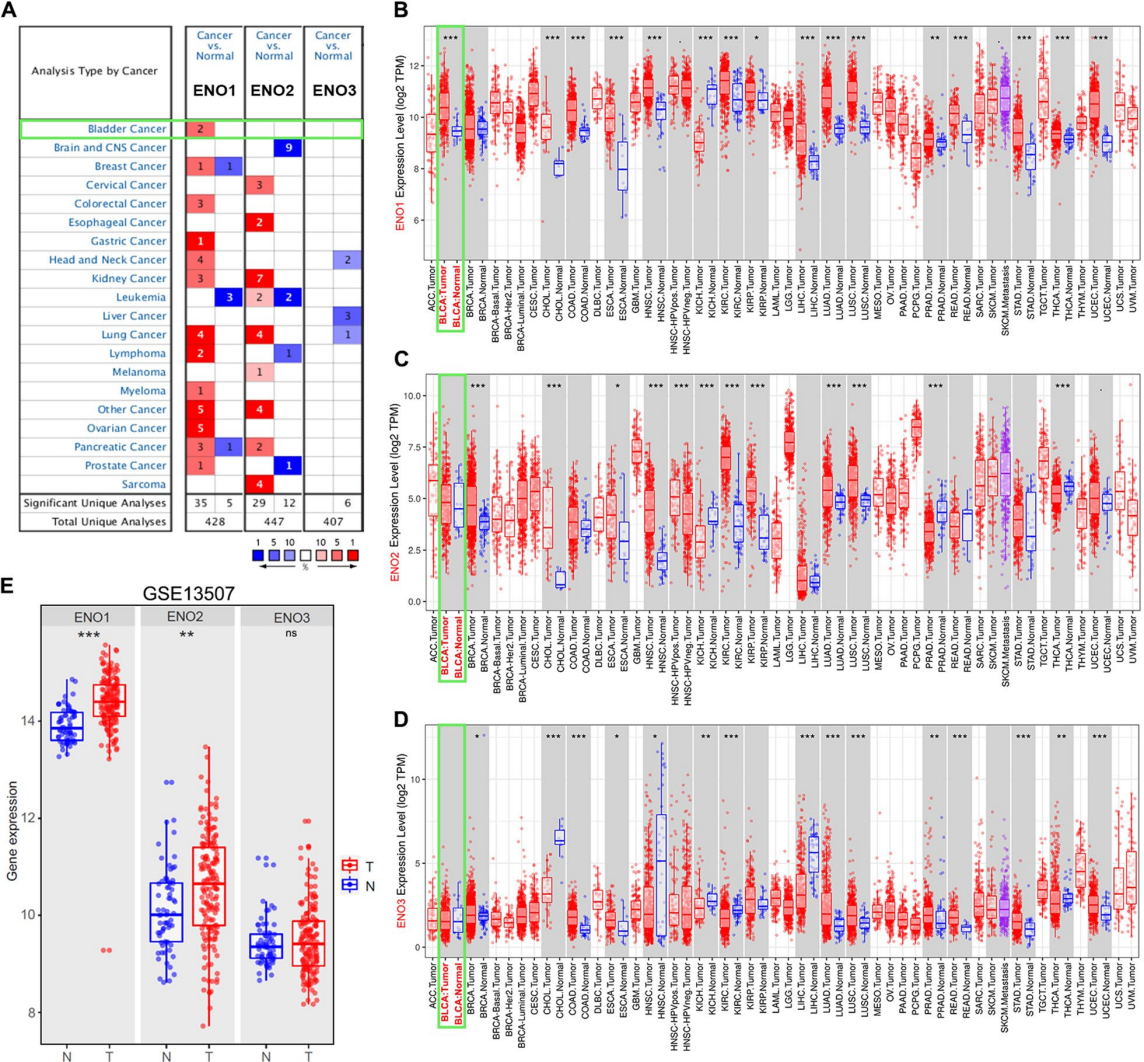
The expression of ENOs in pan-carcinoma and BLCA. **A** Transcriptional expression of ENOs in 20 different types of cancer and noncancerous samples in Oncomine database. **B**–**D** Expression of ENOs in different types of tumor tissues and non-tumor tissues in TIMER database. **E** The mRNA level of ENOs between BLCA tissues (T) and noncancerous counterparts (N) in GSE13507. *p < 0.05, **p < 0.01, ***p < 0.001

To investigate the prognostic worth of ENO isoforms in BLCA patients, we firstly utilized Oncomine database to analyze the expression of ENOs. As shown in Fig. 1A, the mRNA level of ENOs in 20 types of cancers and their normal counterparts were measured, and significantly higher ENO1 mRNA expression was detected in BLCA, while ENO2 and ENO3 expression did not differ as compared to normal samples. Consistently, data from TIMER database manifested that mRNA expression of ENO1, but not ENO2 and ENO3, was upregulated in bladder tumor tissues (Fig. 1B–D). Furthermore, results from GSE13507 revealed that the mRNA level of ENO1 and ENO2 were increased in bladder tumor tissues as compared to normal counterparts (Fig. 1E). In summary, above outcomes highlighted that ENO1 was upregulated in BLCA.

### Correlation of ENOs expression with pathological parameters of BLCA patients

**Fig. 2:**
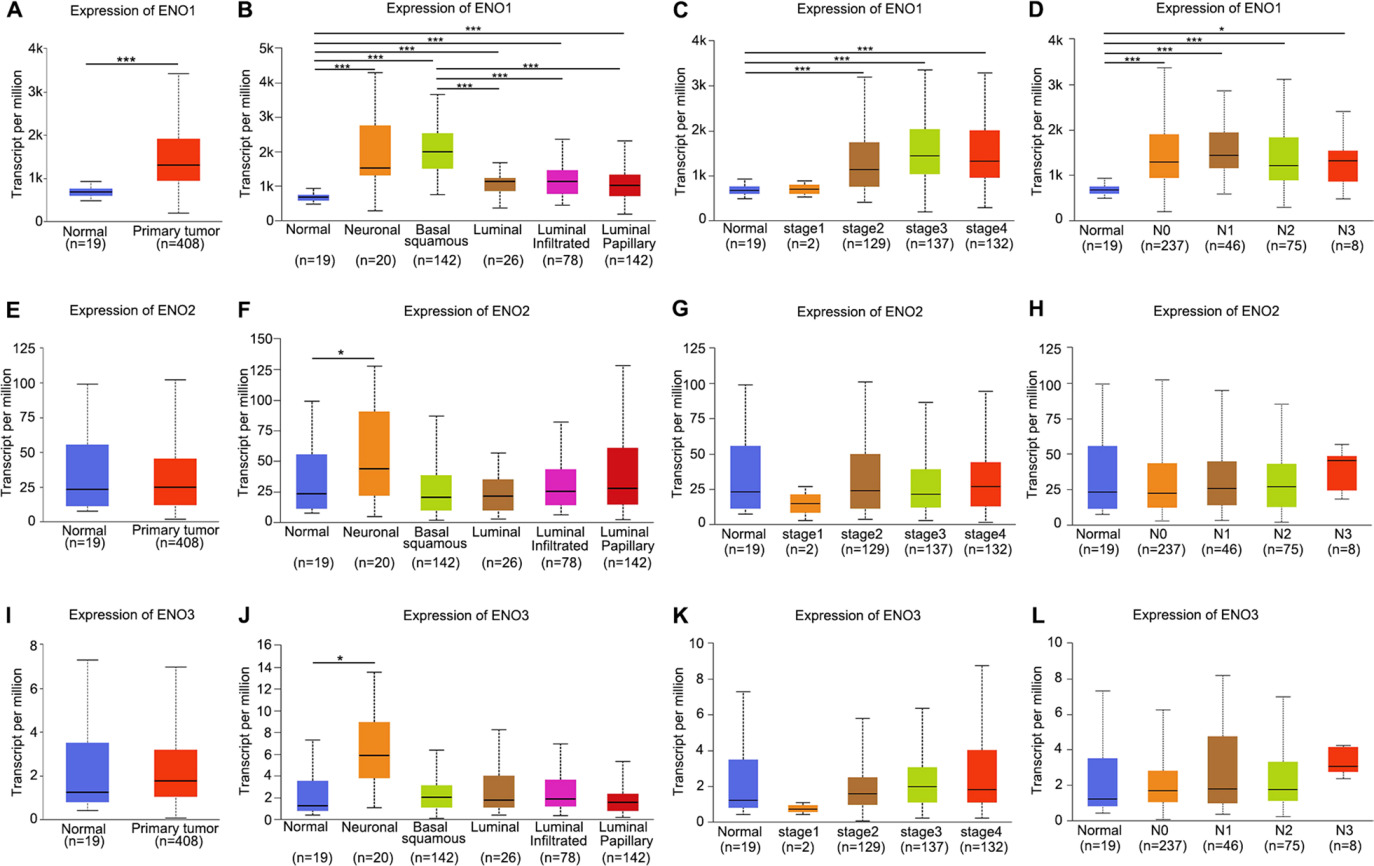
The expression of ENOs among different groups of BLCA patients based on clinical parameters. **A**–**D** Boxplots of ENO1 relative expression based on sample type, molecular subtype, cancer stage and lymph node metastasis. **E**–**H** Boxplots of ENO2 relative expression based on sample type, molecular subtype, cancer stage and lymph node metastasis. **I**–**L** Boxplots of ENO3 relative expression based on sample type, molecular subtype, cancer stage and lymph node metastasis. *p < 0.05, **p < 0.01, ***p < 0.001

Next, UALCAN database was applied to examine the association between ENOs expression and clinicopathologic characteristics in BLCA patients. As shown in Fig. 2A–D, based on the analysis of sample type, molecular subtype, cancer stage and lymph node metastasis, the expression of ENO1 in BLCA patients was dramatically higher than that in normal controls, while there is no significant difference in the expression of ENO2 and ENO3 among the clinical variables (Fig. 2E–L). Moreover, the expression of ENO1 was especially up-regulated in the basal squamous subtype, which was the molecular subtype with poor prognosis in BLCA (Fig. 2B).

### Prognostic value of ENOs in BLCA patients

**Fig. 3:**
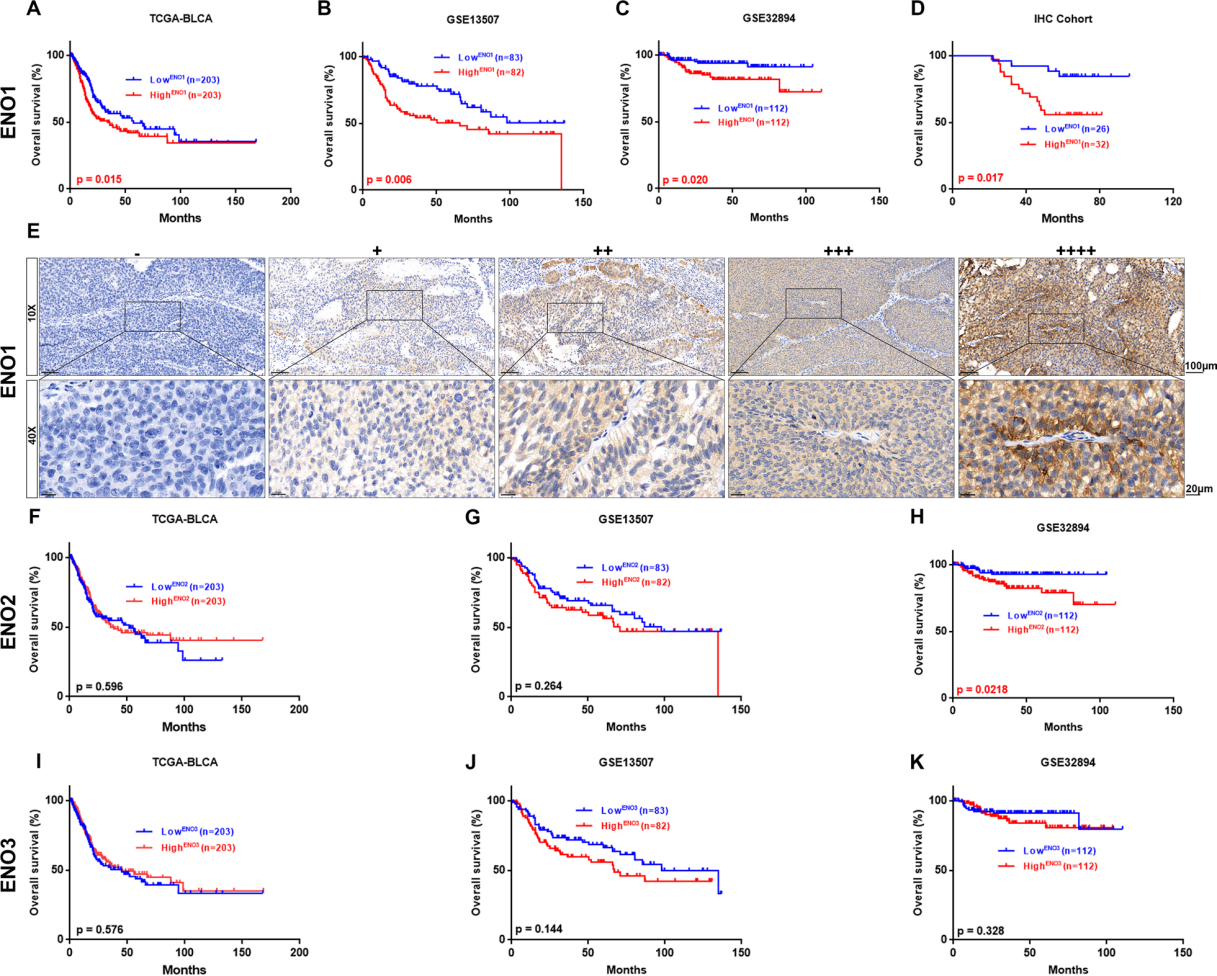
The prognostic significance of ENOs in BLCA patients. Kaplan–Meier curve analysis of overall survival of BLCA patients in TCGA-BLCA (**A**), GES13507 (**B**), GSE32894 (**C**) and IHC cohort (**D**), stratified by ENO1 expression. **E** Representative IHC images of different ENO1 staining intensities in BLCA tissues. Scale bar, 100 and 20 μm. **F**–**K** Kaplan–Meier curve analysis of overall survival of BLCA patients in TCGA-BLCA (**F**, **I**), GES13507 (**G**, **J**) and GSE32894 (**H**, **K**), stratified by ENO2 or ENO3 expression

To further quest the clinical significance of ENOs in BLCA, the correlation of ENOs expression and prognosis was examined by means of Kaplan–Meier survival analysis. The results manifested that ENO1 expression was negatively associated with overall survival in all 3 databases (n = 797 in total, p < 0.05; Fig. 3A–C), while increased expression of ENO2 and ENO3 have no significant correlation with clinical prognosis (Fig. 3F–K). Then, we collected 58 BLCA specimens (IHC cohort) to certify the association of ENO1 expression with prognosis. The representative IHC images for different staining intensities of ENO1 were displayed in Fig. 3E. Consistently, the data from IHC cohort also confirmed that higher ENO1 protein level indicated worse prognosis (p = 0.017, Fig. 3D). Collectively, these results indicated that upregulation of ENO1 predicted unfavorable clinical outcome.

Taken together, the above results shed light on that ENO1 was abnormally overexpressed in BLCA and upregulation of ENO1 predicted unfavorable clinical outcome. These findings implied that ENO1, but not ENO2 and ENO3 among ENOs, may exert significant role in the development of BLCA.

### Overexpression of ENO1 correlated with tumor malignancy of BLCA

**Fig. 4:**
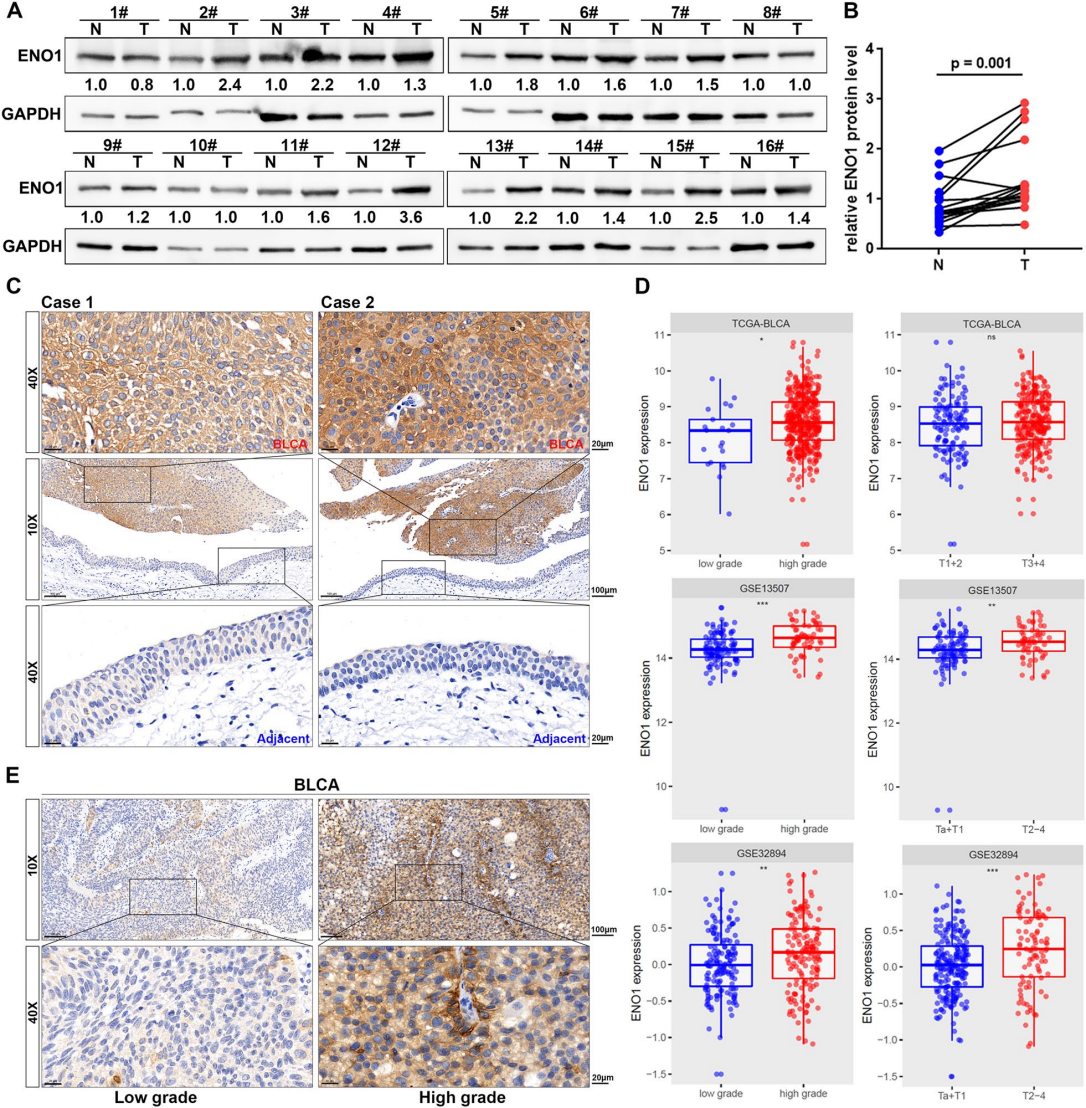
Overexpression of ENO1 correlated with tumor malignancy of BLCA. **A**, **B** ENO1 protein level in 16 paired BLCA tissues (T) and their adjacent normal urothelium tissues (N). **C** Representative IHC images of ENO1 expression in noncancerous and BLCA tissues. Scale bar, 100 and 20 μm. **D** The association of the ENO1 mRNA expression with tumor grade and pathological T stage. **E** Representative IHC images of the ENO1 protein expression in low-grade and high-grade BLCA patients. Scale bar, 100 and 20 μm

To further validate the aberrant ENO1 expression in BLCA, western blotting was performed and increase of ENO1 protein level was detected in 13 out of 16 paired noncancerous and BLCA tissue samples (p = 0.001, Fig. 4A, B). Consistently, IHC staining also displayed that ENO1 notably expressed in the cytoplasm of BLCA cells, while weakly positively stained in normal urothelial cells (Fig. 4C). These findings further demonstrated that ENO1 expression was upregulated in BLCA patients. The association between the ENO1 expression and the clinical features was then explored. The results disclosed that ENO1 expression was remarkably correlated with tumor grade and T stage at both mRNA level (Fig. 4D) and protein level (Table 1). In addition, IHC data displayed the representative images of ENO1 protein expression in patients with low-grade and high-grade (Fig. 4E). These outcomes further proved that ENO1 expression was upregulated in BLCA patients and increased ENO1 expression was significantly correlated with tumor malignancy of BLCA.

**Table 1:** The association between ENO1 protein level and clinicopathological features of BLCA patients (n = 58)

Characteristics	Number	Expression of ENO1		*p* value
		High (n)	Low (n)	
*Gender*				0.356
Male	51	27	24	
Female	7	5	2	
*Age*				0.662
≥ 60	43	23	20	
< 60	15	9	6	
*Tumor grade*				**0.002**
Low	19	5	14	
High	39	27	12	
*T stage*				**0.011**
Ta + T1	34	14	20	
T2-4	24	18	6	
*N stage*				0.243
N0	53	28	5	
≥ N1	5	4	1	
*M stage*				**0.048**
M0	47	23	24	
M1	11	9	2	

Numbers in bold indicate *p* value with statistical difference

### Assessment of ENO1 as a prognostic factor in BLCA patients

**Fig. 5:**
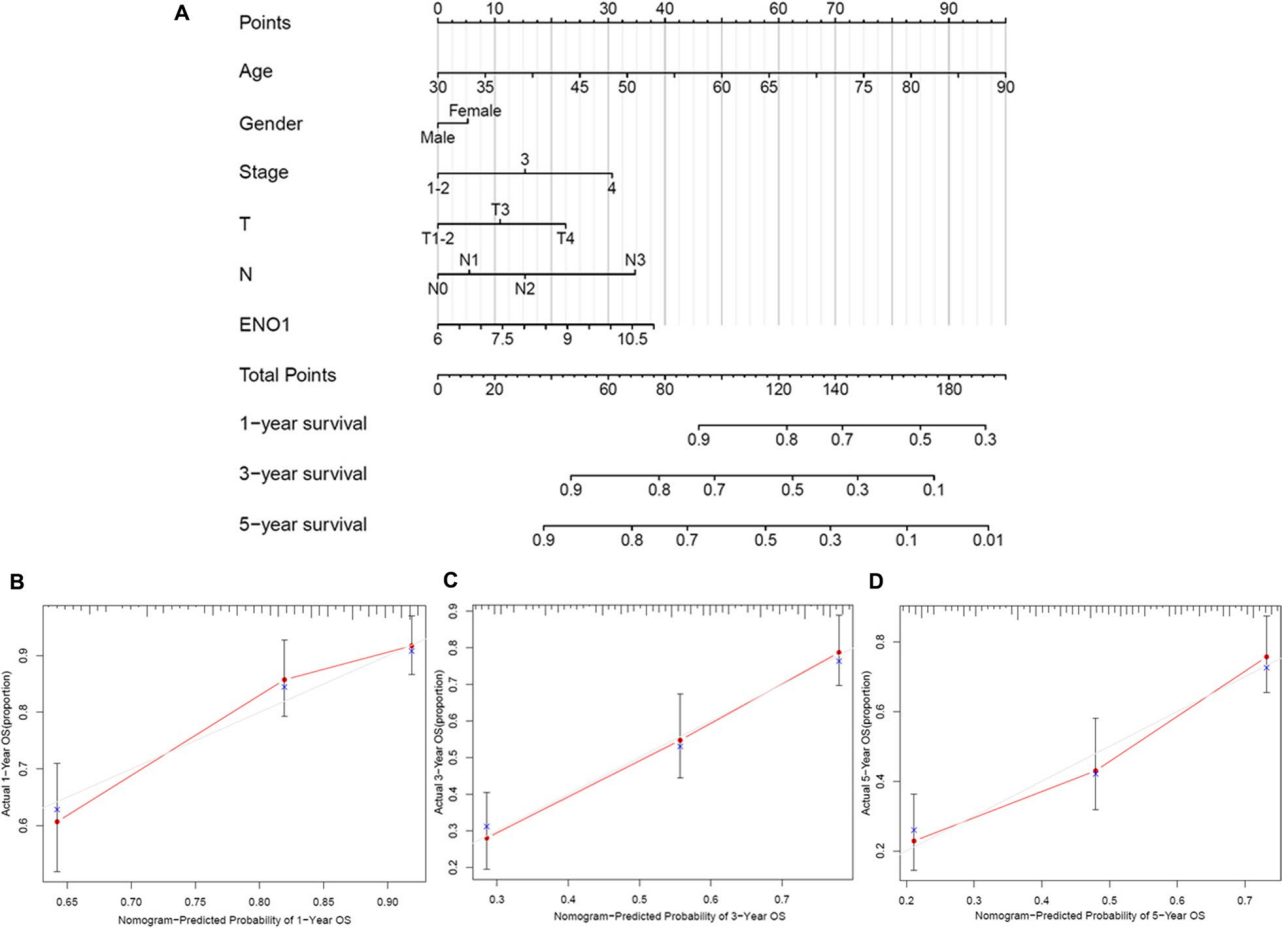
Nomogram analyses of the selected prognostic factors. **A** Nomogram for predicting the 1, 3, and 5-year survival of BLCA patients. The calibration curves of **B** 1-year, **C** 3-year, and **D** 5-year survival of BLCA patients

To further explore the distinct prognostic value of ENO1, a prognostic nomogram was constructed based on TCGA-BLCA by integrating ENO1 expression and clinicopathologic factors (Fig. 5A). The probability of survival for each patient could be easily estimated based on the total score of each variable. As validated by the calibrate curves for the OS probability of 1-year, 3-year, or 5-year, the nomogram exhibited a favorable prognostic effect (Fig. 5B–D). Besides, univariate Cox analysis determined ENO1 as a risk factor and certified that age, T stage and metastasis status could influence the clinical outcome of the patients (Additional file 2: Fig. S1A and B). Furthermore, the ROC curve manifested that ENO1 exerted good performance in anticipating the survival rates of BLCA patients (Additional file 2: Fig. S1C), and subsequent multi-variable time-dependent ROC analysis revealed that ENO1 exhibited higher AUC value relative to other clinicopathological parameters (Additional file 2: Fig. S1D).

### Enrichment analysis of ENO1 gene co-expression network in BLCA

**Fig. 6:**
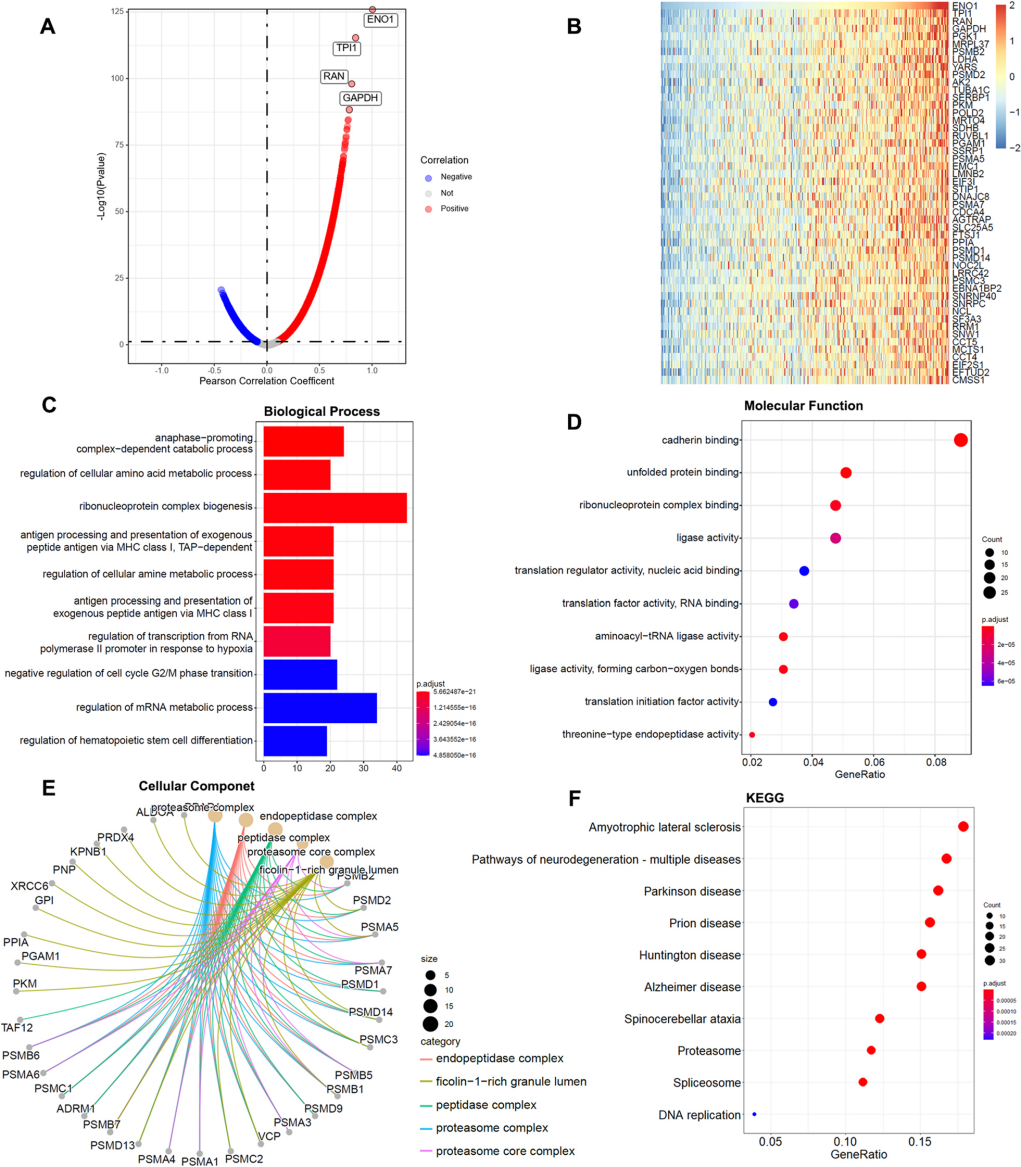
Enrichment analysis of ENO1 gene co-expression network in BLCA. **A** Volcano map of co-expression genes associated with ENO1 expression in TCGA-BLCA. **B** Heat maps of the top 50 co-expression genes correlated with ENO1 expression in TCGA-BLCA. **C**–**E** Enrichment analysis of GO terms for ENO1 co-expression genes. **F** Enrichment analysis of KEGG terms for ENO1 co-expression genes

**Fig. 7:**
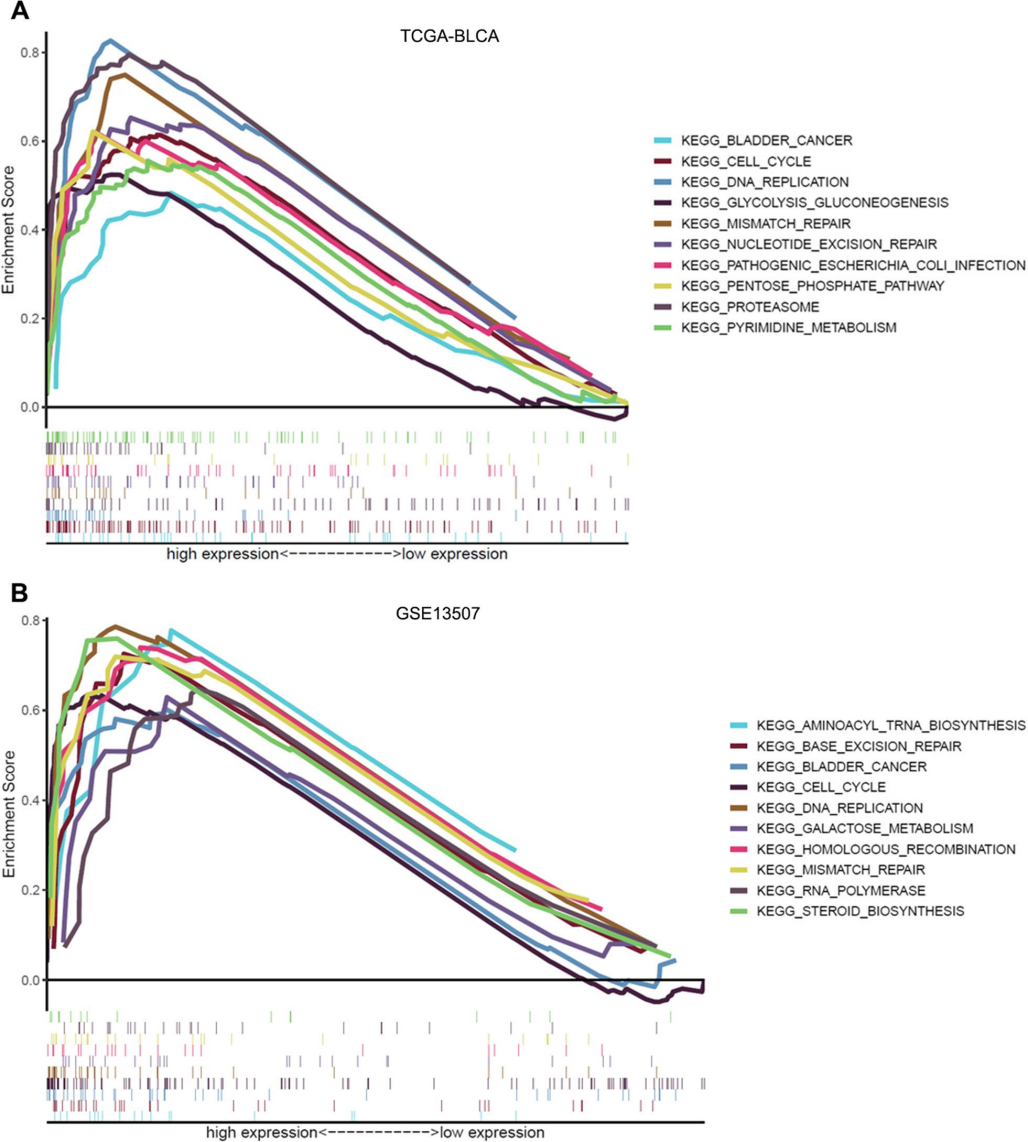
Gene set enrichment analysis of ENO1. **A**, **B** Top ten significant pathways associated with high ENO1 expression in the TCGA-BLCA (**A**) and GSE13507 (**B**)

To gain insight into the function of ENO1 in BLCA, we next performed enrichment analysis. The stat package of R software was firstly utilized to identify genes co-expressed with ENO1 in TCGA-BLCA. As shown in Fig. 6A, 3519 genes were significantly negatively correlated with ENO1 expression, and 16,090 genes were positively correlated with ENO1 expression (p < 0.05). Among which, TPI1 (cor = 0.841, p = 3.984E-116), RAN (cor = 0.804, p = 7.138E-99) and GAPDH (cor = 0.778, p = 3.668E-89) showed the strongest correlation with ENO1. The top 50 genes that were significantly correlated with ENO1 expression were exhibited in Fig. 6B. Then, the top 300 co-expressed genes positively correlated with ENO1 were used for GO function and KEGG pathway enrichment analysis. The top 10 significant terms of Biological Process (BP), Molecular Function (MF), Cell Component (CC) enrichment and KEGG analysis were presented. Notably, GO functional analysis uncovered that ENO1 co-expressed genes were primarily associated with ribonucleoprotein complex biogenesis, cadherin binding and endopeptidase complex (Fig. 6C–E). In addition, KEGG analysis demonstrated that ENO1 co-expressed genes were principally involved in amyotrophic lateral sclerosis, pathways of neurodegeneration-multiple disease and parkinson disease (Fig. 6F). Besides, GSEA results showed that gene sets including cell cycle, bladder cancer and DNA replication were positively enriched in ENO1 high-expression group among TCGA-BLCA and GSE13507 (Fig. 7A and B).

### ENO1 knockdown inhibits cell proliferation and invasion in BLCA

**Fig. 8:**
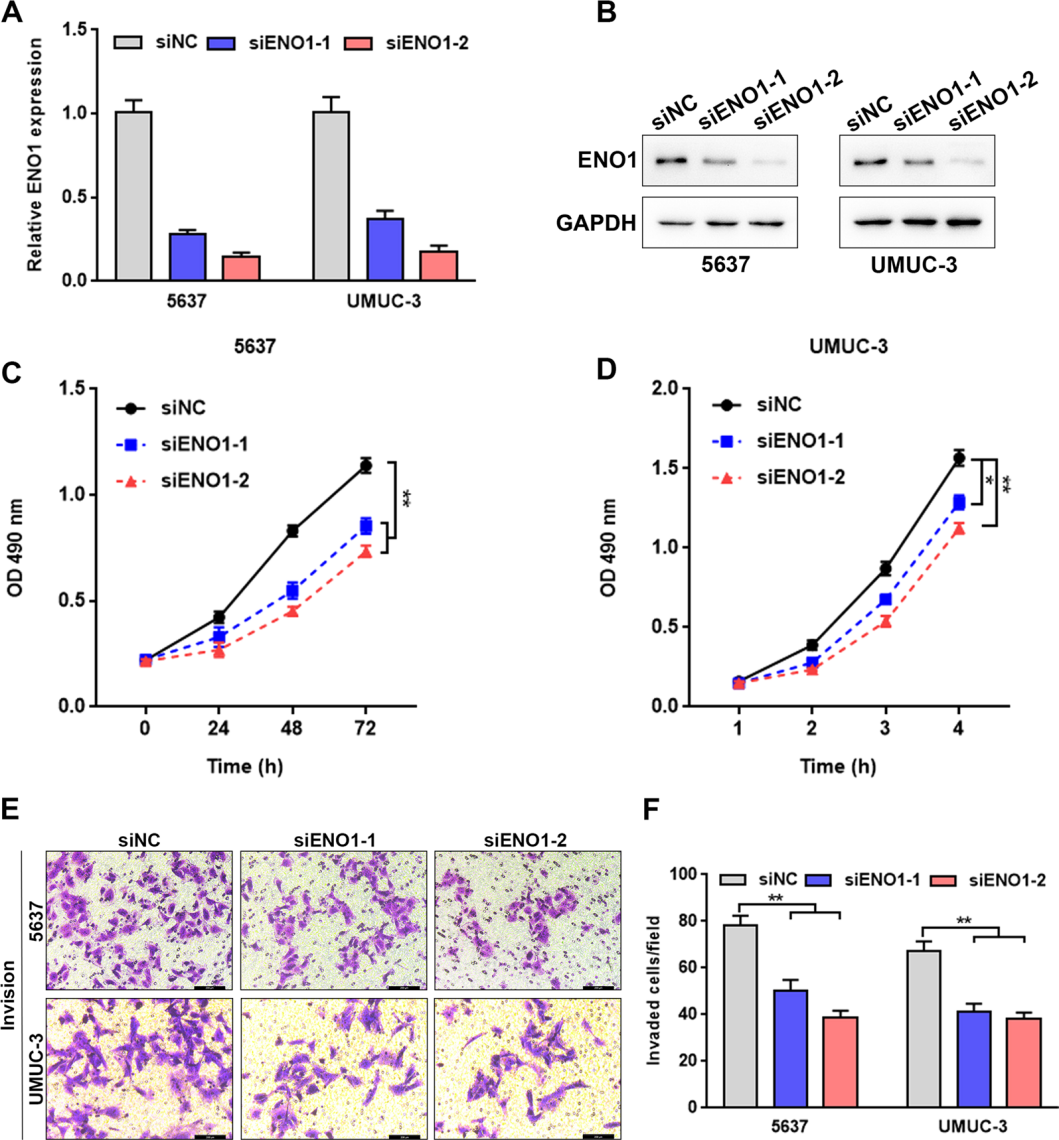
ENO1 knockdown suppresses cell proliferation and invasion**. A**, **B** Transfection efficiency was validated by qRT-PCR and western blotting after transfection with siRNAs in 5637 and UMUC-3 cells. **C**, **D** Effects of ENO1 knockdown on cell viability in 5637 and UMUC-3 cells. **E**, **F** Effects of ENO1 knockdown on cell invasion examined by transwell assay in 5637 and UMUC-3 cells. *p < 0.05, **p < 0.01

Given that the glycolytic activity of ENO1 was closely related to the up-regulation of citrate lyase expression [28], indicating ENO1 might be a promoter of tumor metabolism. Thus, we performed siRNA-mediated loss-of-function approach to better understand the role of ENO1 on BLCA biology. ENO1 knockdown was validated by qRT-PCR and western blotting after ENO1 siRNAs were transfected into 5637 and UMUC-3 cells (Fig. 8A, B). ENO1 deficiency markedly suppressed cells viability of 5637 and UMUC-3 (Fig. 8C, D). Consistently, cells invasive abilities were notably inhibited in ENO1-silenced 5637 and UMUC-3 cells (Fig. 8E, F). Together, these functional data revealed that depletion of ENO1 reduced the capability of BLCA cells to proliferate and invade (Additional file 3).

### ENO1 is involved in tumor immunity in BLCA

Increasing evidence has demonstrated that the tumor microenvironment (TME), which mainly consists of the stromal cells, blood vessels, extracellular matrix and lymphatic networks, plays a significant role in cancer initiation and progression, angiogenesis, and even immune escape [29–31]. The type and proportion of immune cells in the tumor microenvironment are closely related to its physiological state.

**Fig. 9:**
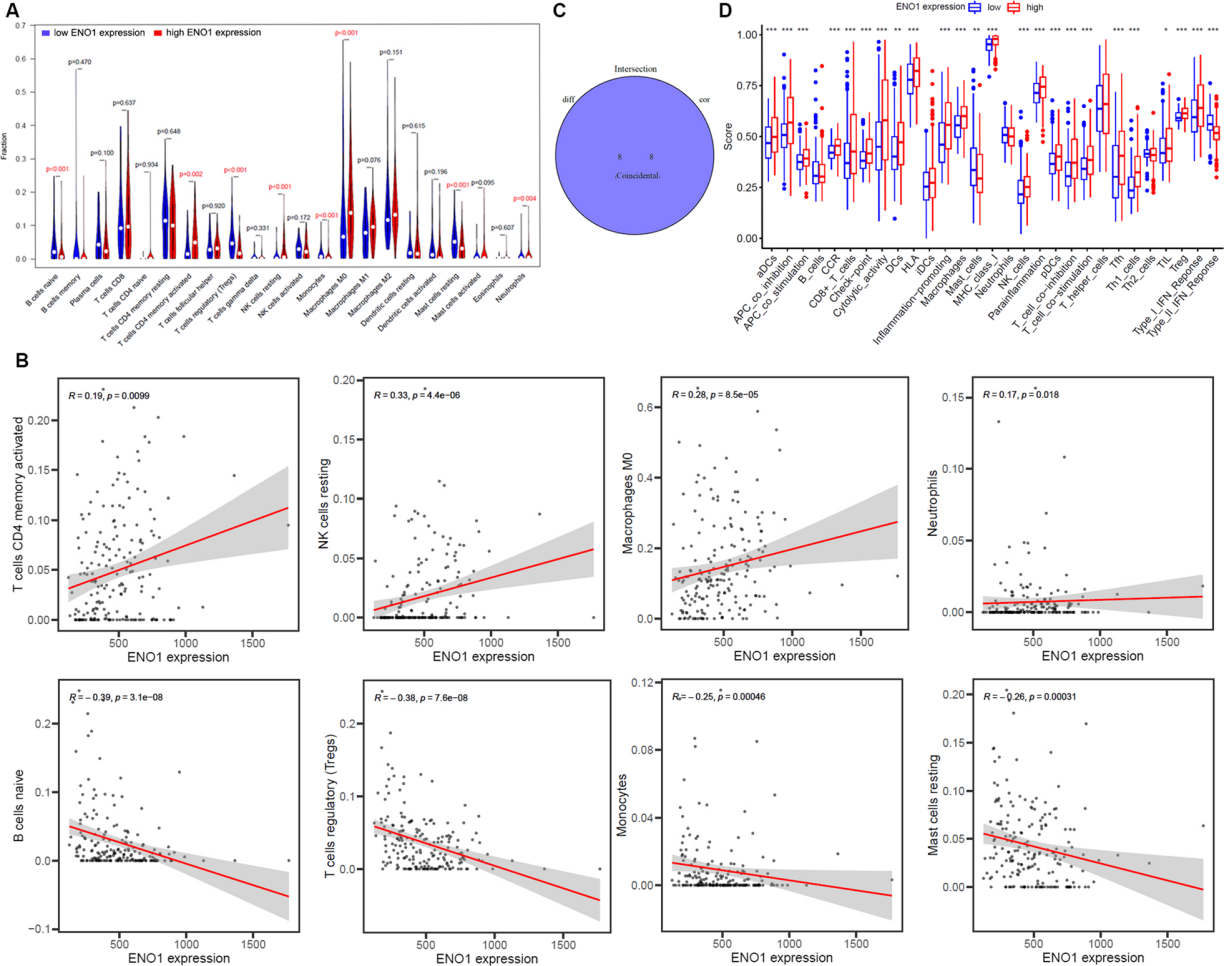
ENO1 is involved in tumor immunity in BLCA. **A** Difference in the proportions of immune cell type in BLCA with low or high ENO1 expression. **B** The correlation between ENO1 expression and the proportions of the 8 most significant tumor-infiltrating immune cells. **C** Venn diagram of intersection between the difference and correlation analyses. **D** The association of ENO1 expression with immune-related pathways or functions. *p < 0.05, **p < 0.01, ***p < 0.001

To examine the interaction of ENO1 in TME, CIBERSORT algorithm was utilized to analyze the difference in the proportions of immune cells in the two groups with high and low ENO1 expression. The results revealed that 8 of 22 immune cell types showed significant differences with ENO1 expression (Fig. 9A). Furthermore, a total of 8 types of immune cells were found to be correlated (four positively and four negatively) with ENO1 expression in the TME of BLCA (Fig. 9B). Unexpectedly, the intersection of the differences and correlation analyses uncovered the two analyses coincide exactly (Fig. 9C). In addition, we also discovered that the ENO1 expression was significantly associated with most immune-related functions or pathways (Fig. 9D), supplying additional evidence for the crucial role of ENO1 in the TME of BLCA.

## Discussion

Bladder cancer (BLCA) is one of the most common malignancies in the genitourinary system [2]. Its high incidence and recurrence rate exhort us to excavate novel biomarkers and therapeutic targets for early diagnosis and treatment [32]. It is widely accepted that aerobic glycolysis is the main way of tumor cell productivity [33]. Therefore, it is possible to inhibit tumor cell aerobic glycolysis by prohibiting the activity of pivotal glycolysis enzymes, thereby suppressing tumor cell proliferation and metastasis. Enolase is the crucial enzyme in the glycolysis pathway, catalyzing the conversion of 2-phosphoglycerate to phosphoenolpyruvate [10, 11]. Therefore, interference with enolase may inhibit the growth of tumor cell by inhibiting the glycolytic pathway, suggesting that enolase has the potential value as therapeutic target.

Hence, to demonstrate the potential worth of ENOs in BLCA, the expression and clinical prognostic value of ENOs were analyzed. First of all, we were pleasantly surprised to find that the expression level of ENO1, but not other ENO isoforms, was significantly up-regulated at the mRNA level in BLCA in Oncomine, TIMER, UALCAN, TCGA-BLCA and GSE13507 databases. Western blotting and immunohistochemical further demonstrated aberrant overexpression of ENO1 at the protein level. Subsequently, the clinical prognostic value of ENO1 was explored. High expression of ENO1 was prominently correlated with high pathological grade and advanced clinical stage. Moreover, overexpression of ENO1 predicted worse prognosis in patients with BLCA. ROC curves also showed that ENO1 had significant diagnostic value for BLCA. Meanwhile, nomogram model illustrated that ENO1 could serve as an independent prognostic factor, which could be utilized to estimate the prognosis of patients. Of particular note, few studies have also elevated the circulating level of ENO1 in cancer patients. For example, ENO1 was overexpressed in the plasma of patients with pancreatic cancer, and the increased plasma ENO1 level was correlated with prognosis and disease progression [34]. In addition, abnormally high circulating ENO1 levels have also been reported in non-small cell lung cancer [35]. Interestingly, plasma ENO1 levels decreased progressively in normal, precancerous condition of the esophagus and esophageal cancer, exactly in contrast to the tissue expression of the protein [36]. These findings implied us further study should be attached to detect the plasma ENO1 level in BLCA patients, which will be of great significance for the translational application of ENO1 in the diagnosis and treatment of BLCA.

The current research on the function of ENO1 in tumor is primarily focused on its effects in glycolysis, while comprehensive analysis of ENO1 in BLCA is less studied. We first identified genes that were significantly correlated with ENO1by constructing a co-expression network, among which TPI1, RAN and GAPDH showed the strongest correlation with ENO1 in BLCA. Previous studies have demonstrated that TPI1 and RAN exerted crucial effects on tumor initiation and progression [37–40]. For example, the reduction of TPI1 in extracellular vesicles mediated by Rab20 downregulation facilitates aerobic glycolysis to drive hepatocarcinogenesis [41]. RAN promotes membrane targeting and stabilization of RhoA to enhance ovarian cancer cell growth and invasiveness [42]. While the function of TPI1 and RAN in BLCA has not been elucidated. Considering the strong correlation of TPI1, RAN with ENO1, and the significant value of ENO1 in BLCA, the role of TPI1 and RAN in BLCA deserves our attention and further exploration. The GO and KEGG function enrichment analyses based on the co-expression network revealed that ENO1 was also involved in many other vital pathways, such as cell cycle and immune-related processes, in addition to regulating glucose metabolism. These findings were consistent with the results of GSEA presented in this study, further reinforcing the effects of ENO1 in regulating cell cycle and immune activity. Additionally, Function experiments demonstrated that ENO1 depletion inhibited cancer cell aggressiveness, further indicating that ENO1 functions as a bad prognostic factor in BLCA.

Increasing evidence has demonstrated that infiltrating immune cells in the tumor microenvironment plays a crucial role in tumorigenesis and progression, thereby affecting the prognosis of tumor patients [43–45]. In this study, we reported that ENO1 expression was significantly correlated with the infiltration of activated memory CD4 cells, resting NK cells, M0 macrophages, neutrophils, naive B cells, regulatory T cells, monocytes, and resting mast cells in BLCA. Moreover, we also identified that ENO1 was involved in multiple immune-related processes, suggesting that ENO1 might exert important regulatory effects in immune-related pathways. Together, these findings indicated that ENO1 might function as a crucial regulator in tumor immunity, as well as a potential biomarker associated with immune infiltration in BLCA. However, the mechanisms involved in how ENO1 affects immune cell infiltration have not been fully elucidated, further in-depth investigation is required to be carried out to elucidate the exact function of ENO1 in the tumor-immune microenvironment.

Changes in tumor metabolism provide new therapeutic targets for tumor therapy. As mentioned earlier, genes related to the glycolytic pathway have been the focus of tumor target research. As the core catalytic enzyme of glycolysis, increasing studies have begun to look for molecules to effectively inhibit ENO1 activity. Previous studies have found that PhAH was a pan-enolase inhibitor, which could effectively inhibit the activity of ENO1, thereby suppressing the growth of pancreatic, breast, and lung cancers [17, 46]. Another study reported that the small molecule AP-III-a4 could directly bind to ENO1 and repress its catalytic activity, thereby prohibiting tumor cell survival without cytotoxicity to normal cells [18, 47]. Taken together, the above research findings and our experimental results have significant guiding help for our clinical research in the future, suggesting that we can manufacture effective inhibitors specific to ENO1 in the future, develop a new tumor treatment strategy for ENO1, and apply to the clinic, which will provide new methods and strategies for clinical treatment of BLCA and even other tumors.

Although the present study initially revealed the association of ENO1 with BLCA, some limitations still exist. Firstly, we confirmed abnormally high ENO1 expression in BLCA at both mRNA and protein levels. However, further studies should be conducted to investigate the specific role and potential molecular mechanisms of ENO1 in tumorigenesis, progression, and immune infiltration. As a metabolite of ENO1 during glycolysis, the biological role of phosphoenolpyruvate in BLCA has also not been fully evaluated, enlightening us that further exploration should be attached to the biological significance of the metabolites correlated with ENO1. In addition, most of the analyses were performed based on TCGA and GEO cohorts, which lack further experimental validation. More in-depth exploration to explain these findings should be systematically interpreted in vitro and in vivo to make the results more convincing.

## Conclusion

Overall, our results revealed that ENO1 was abnormally overexpressed in BLCA, upregulation of ENO1 predicted unfavorable clinical outcomes and was significantly associated with tumor malignancy. These outcomes implied that ENO1 might serve as a promising prognostic biomarker for BLCA. Additionally, ENO1 expression was remarkably related to immune cell infiltration in the TME of BLCA. Taken together, these findings help to deepen our understanding of not only the effects of ENO1 but also its translational application in diagnosis and therapy of BLCA.

## Supplementary Information

**Additional file 1: Table S1.** List for sequences of primer sets and siRNAs.

**Additional file 2: Figure S1.** Prognostic analysis of ENO1 in the BLCA patients. A, B Univariate (A) and Multivariate (B) Cox regression analyses of ENO1 along with clinicopathological characteristics for overall survival in GEO13507. C Time-dependent ROC analysis of ENO1 in estimating the prognostic performance of the BLCA patients in GEO13507. D Multi-variable time-dependent ROC analysis of ENO1 in predicting the overall survival of the BLCA patients in GEO13507.

**Additional file 3: Figure S2.** The originally western blotting images were presented.

## Data Availability

The datasets supporting the conclusions of this article are available in the TCGA and GEO cohorts.
