# Supplementary material for: Identification of ENO1 as a prognostic biomarker and molecular target among ENOs in bladder cancer
Source: J Transl Med. 2022 Jul 14;20:315. doi: 10.1186/s12967-022-03509-1 (PMC9281045; doi:10.1186/s12967-022-03509-1)
Supplement: Supplementary file 1 — Additional file 1: Table S1. List for sequences of primer sets and siRNAs. [file 12967_2022_3509_MOESM1_ESM.docx]

**Table S1 List for sequences of primer sets and siRNAs**

|  | **Direction** | **Sequences (5’-3’)** |
| --- | --- | --- |
| **Primer sets for qRT-PCR** |  |  |
| *β-actin* | Forward | CATGTACGTTGCTATCCAGGC |
|  | Reverse | CTCCTTAATGTCACGCACGAT |
| *ENO1* | Forward | GTACCGCCACATCGCTGACTTG |
|  | Reverse | AGCATGAGAACCGCCATTGATGAC |
| **siRNA sequences** |  |  |
| siENO1-1 | Sense | GCUGCUUACUGUAACUGUAUC |
|  | Antisense | UACAGUUACAGUAAGCAGCUG |
| siENO1-2 | Sense | GGAGUUGGAGACCAGUCUAGC |
|  | Antisense | UAGACUGGUCUCCAACUCCUG |
