# Supplementary figures and images for: Identification of ENO1 as a prognostic biomarker and molecular target among ENOs in bladder cancer
Source: J Transl Med. 2022 Jul 14;20:315. doi: 10.1186/s12967-022-03509-1 (PMC9281045; doi:10.1186/s12967-022-03509-1)

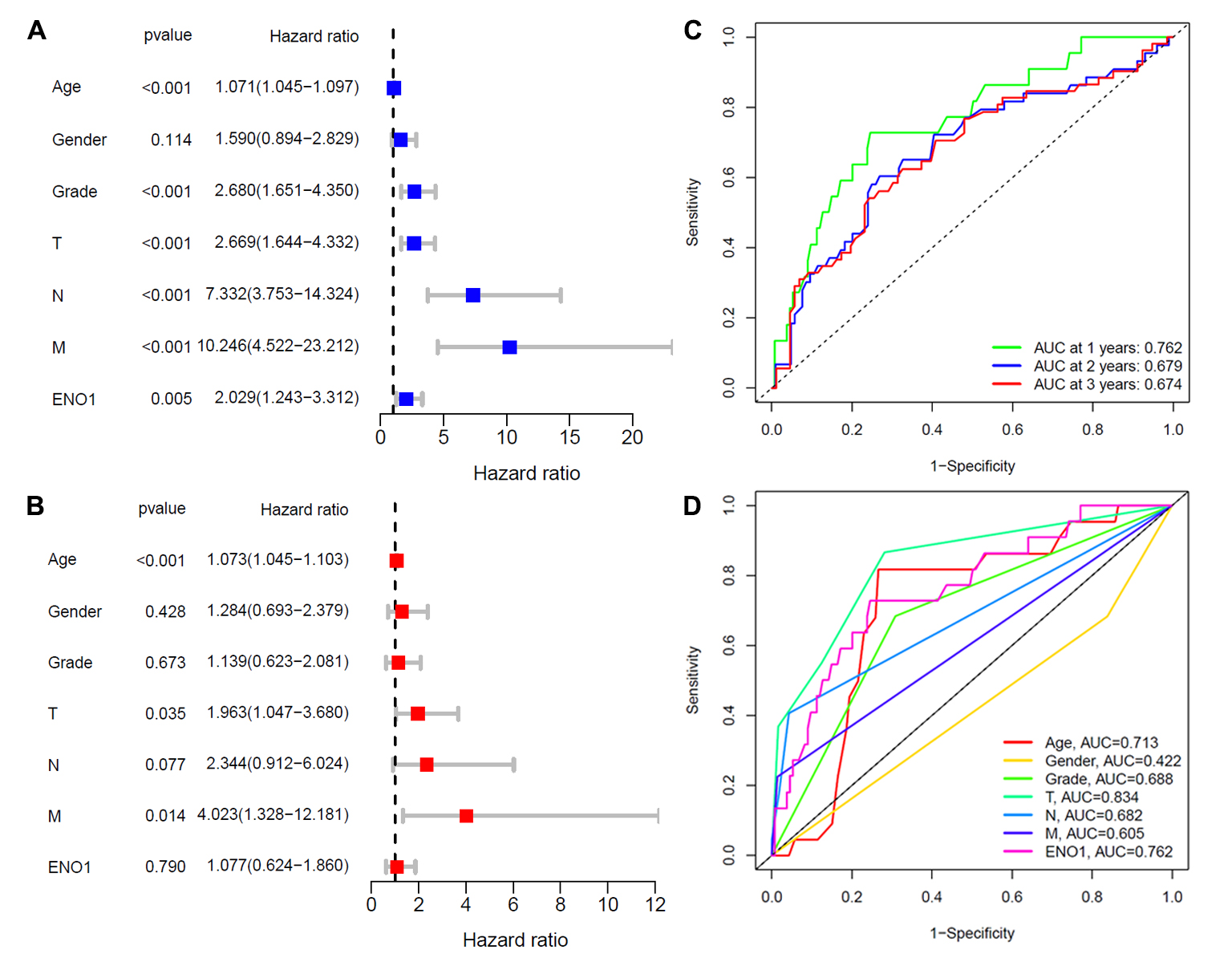

Supplement: Supplementary file 2 — Additional file 2: Figure S1. Prognostic analysis of ENO1 in the BLCA patients. A, B Univariate (A) and Multivariate (B) Cox regression analyses of ENO1 along with clinicopathological characteristics for overall survival in GEO13507. C Time-dependent ROC analysis of ENO1 in estimating the prognostic performance of the BLCA patients in GEO13507. D Multi-variable time-dependent ROC analysis of ENO1 in predicting the overall survival of the BLCA patients in GEO13507. [file 12967_2022_3509_MOESM2_ESM.jpg]

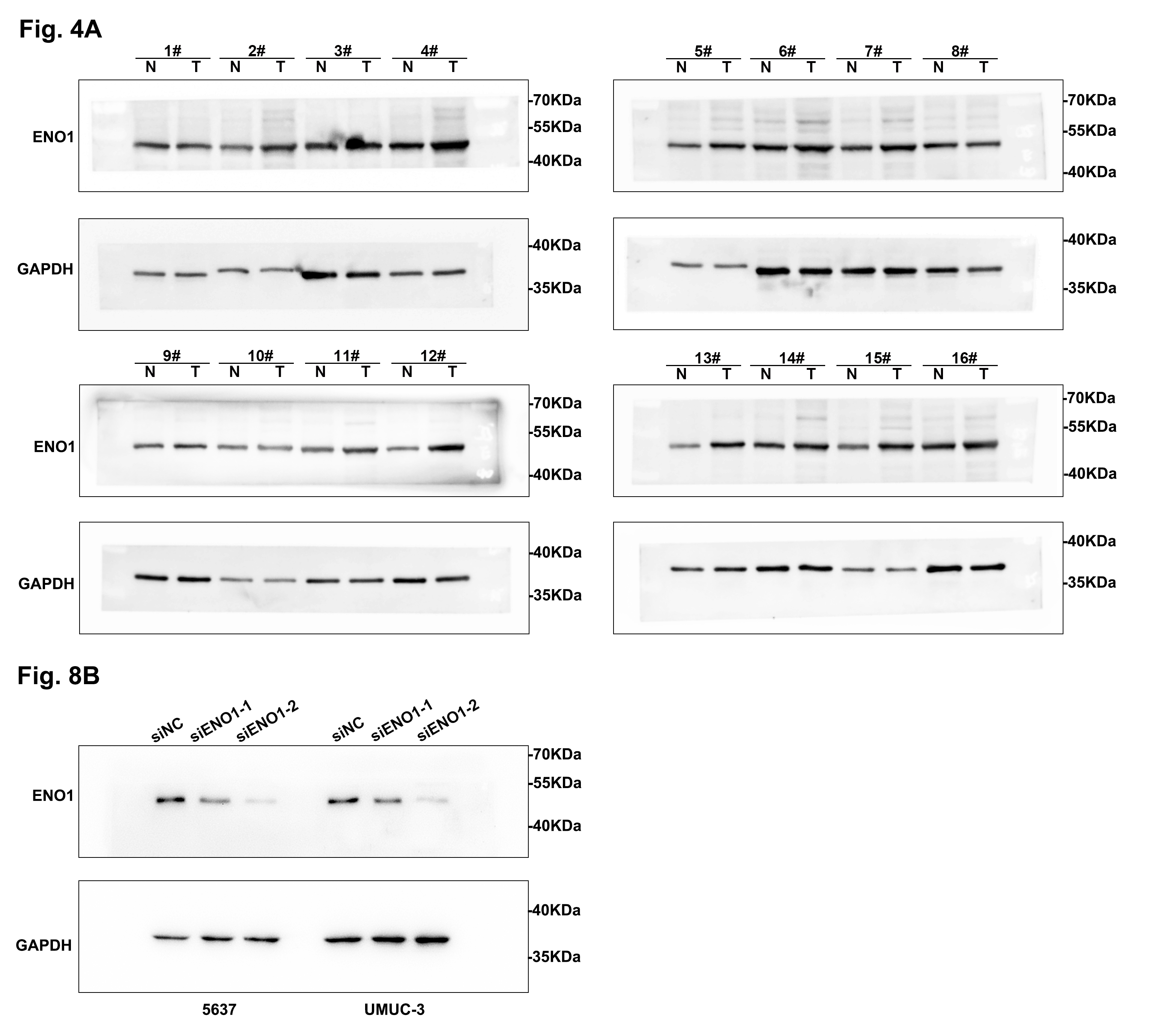

Supplement: Supplementary file 3 — Additional file 3: Figure S2. The originally western blotting images were presented. [file 12967_2022_3509_MOESM3_ESM.jpg]
